# Age-specific global epidemiology of hydrocephalus: Systematic review, metanalysis and global birth surveillance

**DOI:** 10.1371/journal.pone.0204926

**Published:** 2018-10-01

**Authors:** Albert M. Isaacs, Jay Riva-Cambrin, Daniel Yavin, Aaron Hockley, Tamara M. Pringsheim, Nathalie Jette, Brendan Cord Lethebe, Mark Lowerison, Jarred Dronyk, Mark G. Hamilton

**Affiliations:** 1 Division of Neurosurgery, Department of Clinical Neuroscience, University of Calgary, Calgary, Alberta, Canada; 2 Department of Neuroscience, Washington University School of Medicine, St. Louis, Missouri, United States of America; 3 Department of Neurosurgery, Alberta Children’s Hospital, University of Calgary, Calgary, Alberta, Canada; 4 Department of Orthopedic Surgery, New York University, New York, New York, United States of America; 5 Division of Neurology, Department of Clinical Neuroscience, University of Calgary, Calgary, Alberta, Canada; 6 Department of Neurology, Alberta Children’s Hospital, University of Calgary, Calgary, Alberta, Canada; 7 Department of Neurology, Icahn School of Medicine at Mount Sinai, New York, New York, United States of America; 8 Clinical Research Unit, University of Calgary, Calgary, Alberta, Canada; University of Mississippi Medical Center, UNITED STATES

## Abstract

**Background:**

Hydrocephalus is a debilitating disorder, affecting all age groups. Evaluation of its global epidemiology is required for healthcare planning and resource allocation.

**Objectives:**

To define age-specific global prevalence and incidence of hydrocephalus.

**Methods:**

Population-based studies reporting prevalence of hydrocephalus were identified (MEDLINE, EMBASE, Cochrane, and Google Scholar (1985–2017)). Preferred Reporting Items for Systematic Reviews and Meta-Analyses guidelines were followed. Two authors reviewed abstracts, full text articles and abstracted data. Metanalysis and meta-regressions were used to assess associations between key variables. Heterogeneity and publication bias were assessed. Main outcome of interest was hydrocephalus prevalence among pediatric (≤ 18 years), adults (19–64 years), and elderly (≥ 65) patients. Annual hydrocephalus incidence stratified by country income level and folate fortification requirements were obtained (2003–2014) from the International Clearinghouse for Birth Defects Surveillance and Research (ICBDSR).

**Results:**

Of 2,460 abstracts, 52 met review eligibility criteria (aggregate population 171,558,651). Mean hydrocephalus prevalence was 85/100,000 [95% CI 62, 116]. The prevalence was 88/100,000 [95% CI 72, 107] in pediatrics; 11/100,000 [95% CI 5, 25] in adults; and 175/100,000 [95% CI 67, 458] in the elderly. The ICBDSR-based incidence of hydrocephalus diagnosed at birth remained stable over 11 years: 81/100,000 [95% CI 69, 96]. A significantly lower incidence was identified in high-income countries.

**Conclusion:**

This systematic review established age-specific global hydrocephalus prevalence. While high-income countries had a lower hydrocephalus incidence according to the ICBDSR registry, folate fortification status was not associated with incidence. Our findings may inform future healthcare resource allocation and study.

## Introduction

Hydrocephalus encompasses a heterogeneous group of pathologies, characterized by abnormal dilatation of the cerebral ventricles[1]. While untreated hydrocephalus may result in progressive neurologic injury and death, complete resolution of symptoms can be achieved with early diagnosis and surgical intervention.[2] Hydrocephalus can present at any age and is a major cause of mortality and morbidity worldwide.[3, 4] Nevertheless, there is heterogeneity in the reported prevalence and incidence of hydrocephalus, often without reference to age or etiology.[5] Despite the substantial demands it places on patients and healthcare providers, hydrocephalus is under-recognized, and incentives to attract specialized health care providers and researchers in the field are limited. With a four-fold variation in reported rates, accurate resource allocation and planning is challenging, which negatively impacts efforts to improve patient outcomes. Defining the global epidemiology of hydrocephalus is a logical first step to understand its burden. Better epidemiologic information will facilitate recommendations for appropriate research and patient-care resource mobilization. Dewan et. al. recently presented a systematic review and metanalysis of the region-specific global incidence of childhood hydrocephalus.[6] The aim of our study was two-fold: 1) to utilize the International Clearinghouse Centre for Birth Defects Surveillance and Research (ICBDSR) registry to determine the incidence of childhood hydrocephalus and understand the effect of country-specific income level and mandatory folate fortification on the reported incidence rates; and 2) to determine the age- and region-specific global prevalence of hydrocephalus using a systematic review and metanalysis of published reports.

## Methods

### Prevalence of hydrocephalus (systematic review and meta-analysis)

Data on the prevalence of hydrocephalus were obtained through a systematic review and metanalysis of published peer-reviewed population-based articles specific to the epidemiology of hydrocephalus. The Preferred Reporting Items for Systematic Reviews and Meta-Analyses (PRISMA) guidelines were followed.[7] (see S1 Table for checklist).

#### Protocol and registration

A study protocol was registered with the PROSPERO International prospective register of systematic reviews [CRD42017060276].[8]

#### Eligibility criteria

Peer-reviewed studies reporting the prevalence of hydrocephalus between January 1985 and March 2017 were included. Studies that did not report original data in English or French were excluded. To determine study eligibility, two reviewers independently screened abstracts and identified full-texts.

#### Information sources

MEDLINE, EMBASE, Cochrane and Google Scholar databases were searched for human studies using terms specific to the epidemiology of hydrocephalus (see S1 Fig for sample search strategy). The reference list of the included studies as well as review articles were screened to ensure additional relevant studies were not missed. The date of last search was March 1, 2017. Upon discussion with a group of experts in hydrocephalus, additional studies not found in the review were added.

#### Search

A search strategy on the epidemiology of hydrocephalus was developed in consultation with two clinical epidemiologists and a research librarian (S1 Fig).

#### Study selection

Following the removal of duplicate citations, abstracts were screened independently by two reviewers. Abstracts of the included titles underwent a similar review process and all non-population-based studies were excluded. Full text review of these selected articles was carried out, again, by two independent reviewers. Discrepancies between reviewers occurred 8 times at various stages and were settled through discussions with the senior author (MGH).

#### Data collection process

Two reviewers independently extracted data in duplicate, and any discrepancies were evaluated to confirm accuracy. For each study, a standardized form, was used to extract the demographics of the study population, location and number of confirmed hydrocephalus cases, period of data collection, diagnostic criteria, imaging modalities and ancillary tests used to diagnose hydrocephalus, as well as any reported prevalence and confidence intervals. The sources of the collected data (surveys, administrative databases, chart reviews and registries) were also recorded. The prevalence of hydrocephalus was obtained from the number of cases and total population sampled by each study, as was stratification by age, when possible (see S2 Fig for data form).

#### Study heterogeneity and publication bias

Sources of between-study heterogeneity such as the source of primary data collection, year of patient recruitment, year of publication, country of publication, and study quality were explored. Publication bias was visually investigated with funnels plots and were statistically analyzed using the Begg and Egger tests.[9, 10]

#### Study quality

A validated Quality Assessment 8-point Scoring System was used to assess study quality.[11, 12] Each study was assigned a summative quality score (Table 1) ranging from 0 to 8, which was obtained by scoring a point for each of: surveying an entire population or using probability sampling; clearly defining the study population; representativeness of the target population; use of standardized methods for data collection; use of validated criteria to assess for hydrocephalus; outlining response rates and defining non-responses in applicable studies; and for reporting confidence intervals.[12] Only studies that scored 3 or above were deemed eligible for inclusion.

**Table 1:** Studies reporting the prevalence of hydrocephalus and their respective Quality Scores (QS).

Author	Year	QS	Country	Continent	Age Category	Source of Data Collection	Year of recruitment		# of Cases	Pop’n	Prevalence /100k
							From	To			
Del Bigio[13]	1998	3	Canada	North America	Adult	Hospital/clinic chart review	1990	1996	138	1138000	12.1
Klassen et. al.[14]	2011	4	USA	North America	Adult	Registry	1995	2003	41	124,277	33.0
Kumar et. al.[15]	2008	5	Australia	Australia	Adult	Survey	2008	2008	2	478	418.4
Tisell et. al.[16]	2005	7	Sweden	Europe	Adult	Hospital/clinic chart review	1996	1998	891	8854322	10.1
Brean et. al.[17]	2009	4	Norway	Europe	Elderly	Hospital/clinic chart review	2004	2004	48	219478	21.9
Hiraoka et. al.[18]	2008	8	Japan	Asia	Elderly	Survey	1990	2000	5	170	2941.2
Iseki et. al.[19]	2009	7	Japan	Asia	Elderly	Hospital/clinic chart review	2000	2004	6	790	759.5
Iseki et. al.[20]	2014	8	Japan	Asia	Elderly	Hospital/clinic chart review	2000	2010	3	211	1421.8
Jaraj et. al.[3]	2014	7	Sweden	Europe	Elderly	Hospital/clinic chart review	1986	2000	2	834	239.8
Jaraj et. al.[3]	2014	7	Sweden	Europe	Elderly	Hospital/clinic chart review	1986	2000	24	404	5940.6
Kuriyama et. al.[21]	2017	6	Japan	Asia	Elderly	Survey	2012	2012	12900	126470588	10.2
Martin-Laez et. al.[22]	2016	3	Spain	Europe	Adult	Hospital/clinic chart review	2003	2012	14	4681095	0.3
Martin-Laez et. al.[22]	2016	3	Spain	Europe	Elderly	Hospital/clinic chart review	2003	2012	20	253148	7.9
Martin-Laez et. al. [22]	2016	3	Spain	Europe	Elderly	Hospital/clinic chart review	2003	2012	33	256721	12.9
Martin-Laez et. al. [22]	2016	3	Spain	Europe	Elderly	Hospital/clinic chart review	2003	2012	75	241481	31.1
Martin-Laez et. al. [22]	2016	3	Spain	Europe	Elderly	Hospital/clinic chart review	2003	2012	45	325858	13.8
Tanaka et. al.[23]	2012	7	Japan	Asia	Elderly	Survey	1998	2001	1	180	555.6
Tanaka et. al.[23]	2012	7	Japan	Asia	Elderly	Survey	1998	2001	4	174	2298.9
Tanaka et. al.[23]	2012	7	Japan	Asia	Elderly	Survey	1998	2001	2	144	1388.9
Abdullah et. al.[24]	2001	4	Malaysia	Asia	Pediatric	Hospital/clinic chart review	1990	1998	285	537736	53.0
Al Salloum et. al.[25]	2011	3	Saudi Arabia	Asia	Pediatric	Door to Door Survey	2004	2005	14	45682	30.6
Al-Jama et. al.[26]	2001	3	Saudi Arabia	Asia	Pediatric	Hospital/clinic chart review	1992	1997	54	14762	365.8
Baer et. al.[27]	2014	5	USA	North America	Pediatric	Database	2009	2010	27	75899	35.6
Botto et. al.[28]	2013	6	USA	North America	Pediatric	Registry	1983	2006	1271	2779437	45.7
Cavalcanti et. al.[29]	2003	5	Brazil	South America	Pediatric	Hospital/clinic chart review	1987	1998	111	35112	316.1
Cherian et. al.[30]	2016	4	India	Asia	Pediatric	Hospital/clinic chart review	2003	2013	13	36074	36.0
Dai et. al.[31]	2011	5	China	Asia	Pediatric	Database	1996	2009	2376	8991522	26.4
Delshad et. al.[32]	2009	3	Iran	Asia	Pediatric	Hospital/clinic chart review	2005	2007	18	61112	29.5
Egbe et. al.[33]	2015	6	USA	North America	Pediatric	Database	2008	2008	264	1014261	26.0
Fan et. al.[34]	2013	5	China	Asia	Pediatric	Database	2000	2010	50	61762	81.0
Fernell et. al.[35]	1998	5	Sweden	Europe	Pediatric	Registry	1991	1994	75	135710	55.3
Garne et. al.[36]	2010	4	Switzerland	Europe	Pediatric	Registry	1996	2003	86	186922	46.0
Glinianaia et. al.[37]	1999	4	England	Europe	Pediatric	Survey	1985	1996	185	500000	37.0
Gonzalez-Andrade et. al.[38]	2010	3	Ecuador	South America	Pediatric	Database	2001	2007	875	2321489	37.7
Groisman et. al.[39]	2013	6	Argentina	South America	Pediatric	Registry	2009	2012	267	294005	90.8
Guardiola et. al.[40]	2009	3	Brazil	South America	Pediatric	Registry	2000	2005	20	26588	75.2
Hannon et. al.[41]	2012	6	England	Europe	Pediatric	Hospital/clinic chart review	1994	2008	695	454080	153.1
Harmat et. al.[42]	2001	3	Hungary	Europe	Pediatric	Hospital/clinic chart review	1990	1998	198	46858	422.6
Jeng et. al.[43]	2011	7	USA	North America	Pediatric	Database	1991	2000	2608	5353022	48.7
Mahmoud et. al.[44]	2014	5	Sudan	Africa	Pediatric	Hospital/clinic chart review	2011	2013	20	5000	400.0
Movafagh et. al.[45]	2008	3	Iran	Asia	Pediatric	Hospital/clinic chart review	2000	2004	21	33380	62.9
Msamati et. al.[46]	2000	3	Malawi	Africa	Pediatric	Hospital/clinic chart review	1998	1999	6	25562	23.5
Munch et. al.[47]	2012	6	Denmark	Europe	Pediatric	Registry	1978	2008	2194	1928683	113.8
Murshid et. al.[48]	2000	4	Saudi Arabia	Asia	Pediatric	Hospital/clinic chart review	1996	1997	26	16550	157.1
Nakling et. al.[49]	2005	4	Norway	Europe	Pediatric	Hospital/clinic chart review	1989	1999	9	18181	49.5
Nogueira et. al.[50]	1992	3	Qatar	Asia	Pediatric	Hospital/clinic chart review	1986	1989	48	41195	116.5
Ogunyemi et. al.[51]	2000	3	USA	North America	Pediatric	Hospital/clinic chart review	1996	1998	4	6877	58.2
Persson et. al.[52]	2005	3	Sweden	Europe	Pediatric	Hospital/clinic chart review	1989	1998	124	253378	48.9
Persson et. al.[53]	2007	3	Sweden	Europe	Pediatric	Hospital/clinic chart review	1999	2002	54	82016	65.8
Rajab et. al.[54]	1998	3	Oman	Asia	Pediatric	Hospital/clinic chart review	1992	1995	106	242764	43.7
Sethna et. al.[55]	2011	7	United Kingdom	Europe	Pediatric	Registry	1994	2008	267	454080	58.8
Shawky et. al.[56]	2011	7	Egypt	Africa	Pediatric	Registry	1995	2009	677	660280	102.5
Sun et. al.[57]	2011	5	China	Asia	Pediatric	Hospital/clinic chart review	1998	2009	77	83888	91.8
Synnes et. al.[58]	2004	3	Canada	North America	Pediatric	Hospital/clinic chart review	1996	1997	67	19507	343.5
Tang et. al.[59]	2006	5	USA	North America	Pediatric	Registry	1996	2000	732	972694	75.3
Waller et. al.[60]	2000	6	USA	North America	Pediatric	Registry	1995	1995	32	111,902	28.6
Xie et. al.[61]	2016	6	China	Asia	Pediatric	Hospital/clinic chart review	2005	2014	702	925413	75.9
Zhang et. al.[62]	2012	5	China	Asia	Pediatric	Hospital/clinic chart review	2005	2008	62	61992	100.0
El Awad.[63]	1992	4	Saudi Arabia	Asia	Pediatric	Hospital/clinic chart review	1988	1990	37	74923	49.4

#### Summary measures

Hydrocephalus was defined as radiographic evidence of ventriculomegaly with correlating clinical symptoms of the syndrome, and papers that did not specify this were excluded[1]. Hydrocephalus associated with spina bifida was recorded separately. New ventricular shunting surgery and ETV done for treatment of hydrocephalus were accepted as surrogate indicators for the identification of hydrocephalus. Revision shunt surgeries and revision ETV’s were excluded. The number of reported cases and the population assessed were analyzed in each reported population group for the prevalence of hydrocephalus per study. As prevalence is a proportion, study estimates were combined using a log transformation to normalize the data.

#### Synthesis of results (prevalence analysis)

The prevalence of hydrocephalus was analyzed for specific age groups: pediatric (perinatal to age 18), adults (age 19 to 64), and elderly (age 65 and above) and by continent. The prevalence models were further stratified by country, continent and paper quality score. To assess for significant between-study heterogeneity the Cochrane Q statistic was calculated and I^2^ was used to quantify between-study heterogeneity. Given disparate study methods and populations sampled, a random effects model was used to obtain a pooled prevalence per 100,000 people with a 95% confidence interval. Confidence intervals were calculated using the Clopper-Pearson or “exact” binomial method. The prevalence of hydrocephalus in spina bifida, which has been quoted as approximately 80% in the literature, was used to adjust the estimates of hydrocephalus in the pediatric population.[64, 65] To that effect, in addition to analyzing hydrocephalus-only cases, separate analyses were performed where 80% of spina bifida cases was added to the hydrocephalus cases prior to performing pooled analyses. Several sub-group analyses were done. All statistical analyses were carried out in *R* version 2.14[66]. Prevalence was reported as rates per 100,000. *P*-value 0.05 was considered significant.

#### Risk of bias across studies

To ensure internal consistency and to permit accurate comparisons, studies examining similar populations, similar diagnoses, using similar methods were grouped together. The *meta* package for R was used to produce the pooled estimates, forest plots, and publication bias assessment[66]. The *metafor* package for R was used to conduct the meta-regression using restricted maximum likelihood estimation[66].

### Incidence of hydrocephalus (ICBDSR registry)

Data on the incidence of hydrocephalus was obtained from the ICBDSR’s annual reports (Table 2).[67] The ICBDSR is a non-governmental organization affiliated with the World Health Organization that collects data on birth defects including hydrocephalus and spina bifida from 42 surveillance programs, spanning 36 countries. The ICBDSR reporting guidelines stipulates cases of hydrocephalus diagnosed at birth to be reported separate from hydrocephalus associated with spina bifida. Spina bifida includes meningocele, meningomyelocele, myelocele, myelomeningocele and rachischisis, but excludes spina bifida occulta.[67] Of note, cases of postnatally acquired hydrocephalus are not reported to the ICBDSR. For this study, the annual incidence of hydrocephalus diagnosed at birth and spina bifida in the most recent 11 years (2003–2014) were retrieved from the database, except for 2008 where no data were available.

**Table 2:** Annual incidence of hydrocephalus reported by birth surveillance registries.

Country	Program	Folate legislation (Year)	Income Level	2014	2013	2012	2011	2010	2009	2007	2006	2005	2004	2003
Argentina	RENAC	M (2002)	Middle	111.26	*	184.4	*	*	*	*	*	*	*	*
Australia	WARDA	M (2009)	High	20.53	21.76	32.39	38.44	45.64	39.9	47.81	62.68	40.5	52.49	*
Australia	VBDR	M (2009)	High	*	*	*	*	77.38	73.29	69.01	70.64	66.09	77.69	80.45
Canada	Alberta ACASS	M (1998)	High	59.25	65.13	41.21	50.56	59.39	71.86	47.78	54.25	59.79	44.12	58.69
Canada	British Columbia	M (1998)	High	*	*	*	*	62.81	56.98	61.01	36.69	29.58	47.26	*
Canada	CCASS	M (1998)	High	57.61	65.1	61.84	62.6	46.42	54.78	61.76	56.77	*	84.82	29.52
Chile	RRMC-SSM	M (1996)	High	*	*	29.31	36.63	14.43	29.79	38.08	45.78	48.26	21.91	*
China	CBDMN	NM	Middle	*	*	*	*	*	*	*	31.8	36.31	39.99	39.75
China	BDSS-Beijing	NM	Middle	*	*	*	*	*	*	7.36	8.88	10.88	11.99	16.86
Colombia	BCMSP	M (1996)	Middle	*	30.49	42.96	49.2	*	*	*	*	*	*	*
Costa Rica	CREC	M (1997)	Middle	81.31	81.31	29.43	42.39	53.49	*	30.75	54.83	*	36.55	*
Cuba	RECUMAC	M (2012)	Middle	*	13.38	18.58	15.97	19.92	7.45	27.98	31.26	18.25	27.94	*
Czech Republic		NM	High	32.12	24.96	30.65	26.12	26.7	29.81	18.54	28.59	50	39.76	28.58
Finland		NM	High	*	*	39.24	37.93	46.89	39.03	39.71	32.79	61.61	55.59	39.01
France	REMERA	NM	High	73.33	49.02	38.28	18.62	37.38	36.37	27.11	32.16	31.46	28.04	29.72
France	Paris	NM	High	65.72	75.82	76.64	114.05	112.94	121.49	89.02	68.3	93.99	74.94	67.53
France	Strasbourg	NM	High	*	*	*	53.34	*	35.97	7.27	14.92	7.41	14.84	0
Germany	Saxony-Anhalt	NM	High	29.5	59.15	51.83	29.05	45.04	28.62	46.43	57.43	29.45	79.15	60.61
Hungary		NM	High	82.46	16.15	44.09	53.63	66.28	31.6	33.67	30.33	29.42	29.8	24.59
India	BDRI	NM	Low	*	61.5	54.12	67.42	91.59	*	*	*	*	*	*
Iran	TRoCA	M (2007)	Middle	130.04	204.62	68.02	99.16	92.22	131.65	135.25	68.45	*	*	*
Ireland		NM	High	*	*	36.19	45.08	34.82	34.13	21.33	45.99	29.62	35.09	69.35
Israel	IBDMS	NM	High	*	47.25	36.55	60.64	50	29.25	54.16	72.02	53.75	64.89	70.34
Italy	BDRCam	NM	High	*	*	25.72	53.6	58.32	7.14	13.43	8.21	8.35	14.35	36.21
Italy	LBDR	NM	High	12.6	*	*	*	*	*	*	25.15	24.45	62.22	47.2
Italy	IMER	NM	High	*	*	47.45	28.06	23.59	9.84	23.93	21.46	40.13	38.45	33.8
Italy	North East	NM	High	*	15.74	*	2.19	*	10.97	26.75	3.85	14.44	22.61	12.05
Italy	RTDC	NM	High	13.33	19.88	35.67	16.36	9.64	9.69	20.4	20.7	50.93	7.52	7.58
Italy	CMLR	NM	High	*	24.46	69.97	29.67	17.32	*	*	*	*	*	*
Japan	JAOG	NM	High	89.74	117.13	116.38	118.15	121.13	106.8	96.91	97.11	129.96	108.68	109.87
Malta		NM	High	*	23.2	24.78	71.77	94.61	128.27	77.62	102.51	51.02	52.27	77.26
Mexico	RYVEMCE	M (1999)	Middle	114.23	61.1	62.67	81.23	75.69	115.73	101.82	122.07	98.97	100.78	87.98
Mexico	BDSP	M (1999)	Middle	47.89	*	*	*	*	*	*	*	*	*	*
New Zealand		NM	High	24.36	53.38	52.86	44.5	52.43	46.5	51.63	49.53	42.49	54.02	57.02
Netherlands		NM	High	12.06	34.91	51.23	27.55	16.73	45.25	48.59	36.59	34.99	34.26	39.05
Norway	MBRN	NM	High	*	37.32	63.86	44.22	30.7	50.53	51.8	51.64	66.23	31.93	47.11
Russia	MRRCM	NM	Middle	*	*	*	28.12	46.28	50.65	35	50.71	55.45	57.65	51.01
Saudi Arabia	MSD-BDR	M (2000)	High	*	178.2	120.55	*	*	*	*	*	*	*	*
Slovak Republic		NM	High	62.82	36.06	34.65	37.43	31.26	32.95	45.77	53.75	50.07	*	*
South Africa	SABDSS	M (2003)	Middle	*	*	*	*	*	*	*	*	85.32	*	*
Spain	ECEMC	NM	High	14.25	24.19	21.82	18.35	21.67	14.63	29.98	26.92	18.43	21.63	35.78
Sweden		NM	High	*	16.95	14.85	25.29	27.55	23.6	16.72	24.69	30.15	30.16	26.14
Ukraine	OMNI-Net	NM	Low	65.79	97.43	82.42	83.11	87.93	78.02	71.39	83.3	98.25	84.27	68.7
United Kingdom	CARIS	NM	High	67.76	47.53	74.71	56.95	58.63	43.37	30.52	49.22	*	*	*
United Kingdom	WANDA	NM	High	*	*	*	*	27.16	31.03	*	*	*	*	*
United Kingdom	England & Wales	NM	High	*	*	*	*	*	*	14.02	15.24	16.32	16.69	17.24
USA	ARHMS	M (1996)	High	72.73	*	*	*	*	*	*	*	*	*	*
USA	MACDP	M (1996)	High	*	*	123.41	116.53	78.33	68.18	72.99	55.39	49.77	72.3	79.92
USA	IRCID	M (1996)	High	*	*	*	*	*	*	*	*	*	*	*
USA	BDES	M (1996)	High	109.38	109.38	91.86	91.37	90.99	79.6	89.77	70.33	68.06	*	*
USA	UBDN	M (1996)	High	28.99	29.17	20.97	33.24	55.44	72.31	48.28	45.17	*	*	*
United Arab Emirates		NM	High	*	*	*	*	*	*	*	*	102.45	178.51	179.3

M = mandatory

NM = non-mandatory folate legislation

Income level is based on World Bank 2015 Gross National Product income level designation

* Data not reported by the surveillance program for the corresponding year

#### Income level

The correlation between income level and incidence of hydrocephalus was analyzed. The World Bank’s 2015 fiscal year data were used to categorize countries into low, medium and high levels of income based on their 2015 Gross National Income (GNI) per capita: low income (less than $1,025), middle income (from $1,026 to $12,475) and high income (greater than $12,475).[68] The low and medium income groups were combined and compared to the high income group of countries.

#### Mandatory folate fortification

The effect of folate fortification on the incidence of spina bifida is an ongoing global debate, with variable results reported among studies.[69] Given the high prevalence of spina bifida-associated hydrocephalus, estimates of hydrocephalus incidence were stratified by country mandatory folate fortification status. Each country’s folate fortification status was obtained from the Food Fortification Initiative, a multinational collaboration aimed to improve health through industrial fortification of grain products.[70] Countries were stratified into mandatory vs non-mandatory fortification depending on the presence or absence of legislation that mandates the fortification of one or more types of wheat or maize flour or rice with folic acid.[70]

#### Incidence analysis

Incidence of hydrocephalus was defined as new cases per year reported by the respective surveillance programs. Mean annual incidences were obtained as pooled estimates of the reported incidences per country for each year. The incidences were further stratified by continent. Correlations between incidence and income-levels, and mandatory folate fortification status were analyzed. Similar to prevalence, in addition to analyzing hydrocephalus-only cases, separate analyses were performed where 80% of spina bifida cases was added to the hydrocephalus cases prior to performing pooled analyses.[64, 65] Incidence was reported as rates per 100,000. Confidence intervals of 95% were calculated using the Clopper-Pearson method. *P*-value of 0.05 was considered significant. All statistical analyses were carried out in *R* version 2.14[66].

## Results

**Fig 1:**
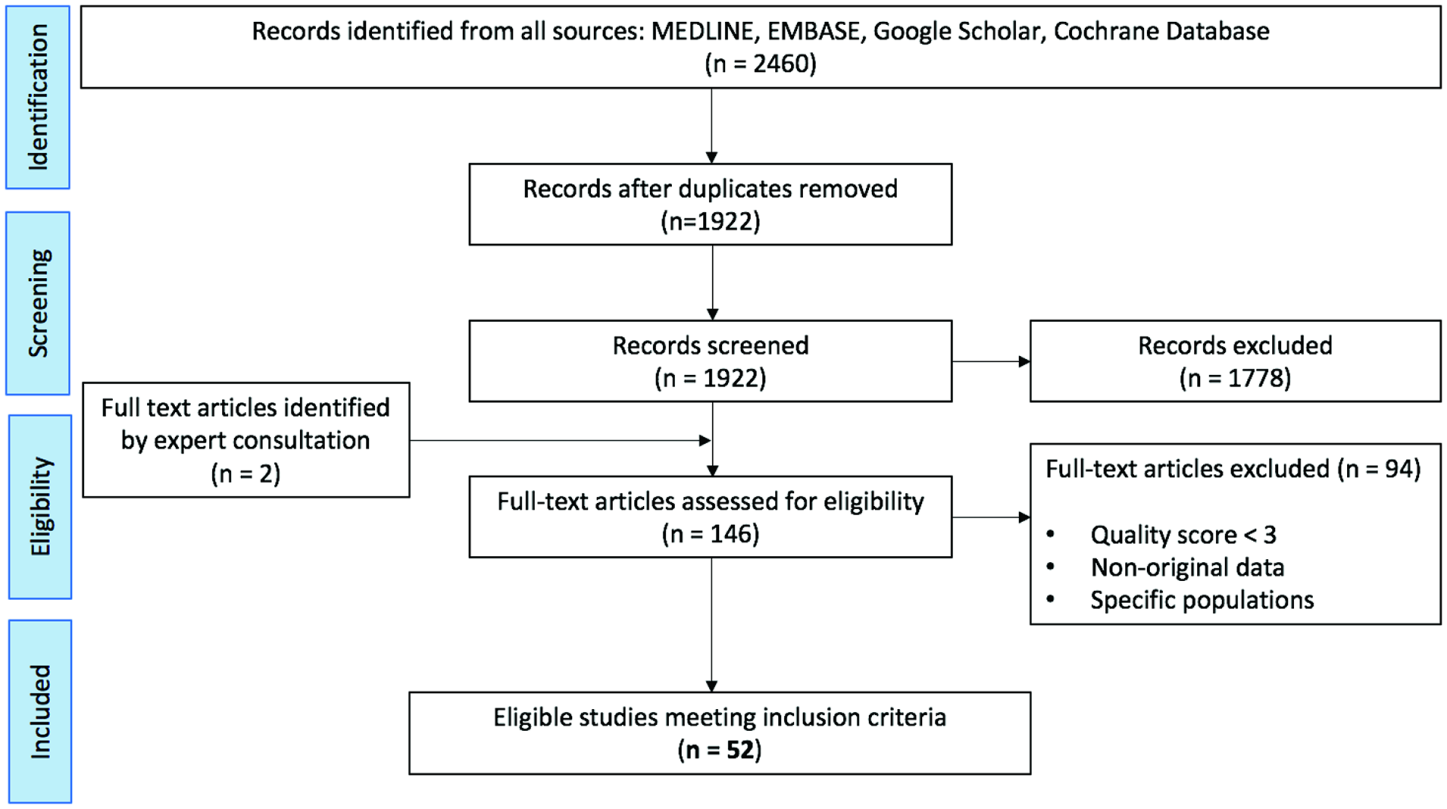
PRISMA flowchart of a systematic review of the global epidemiology of hydrocephalus.

**Fig 2:**
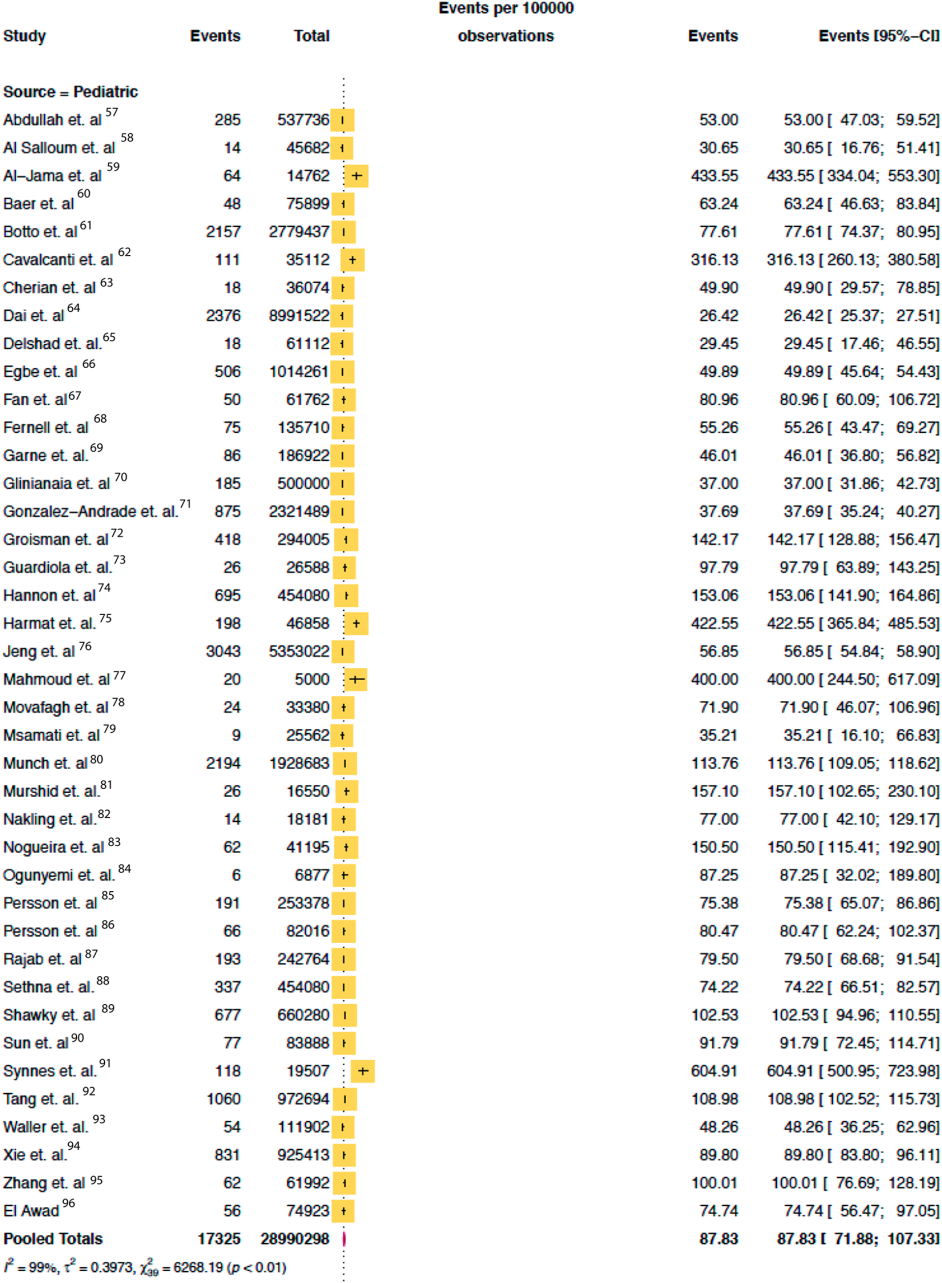
Pooled mean prevalence/100,000 of hydrocephalus in pediatric population.

**Fig 3:**
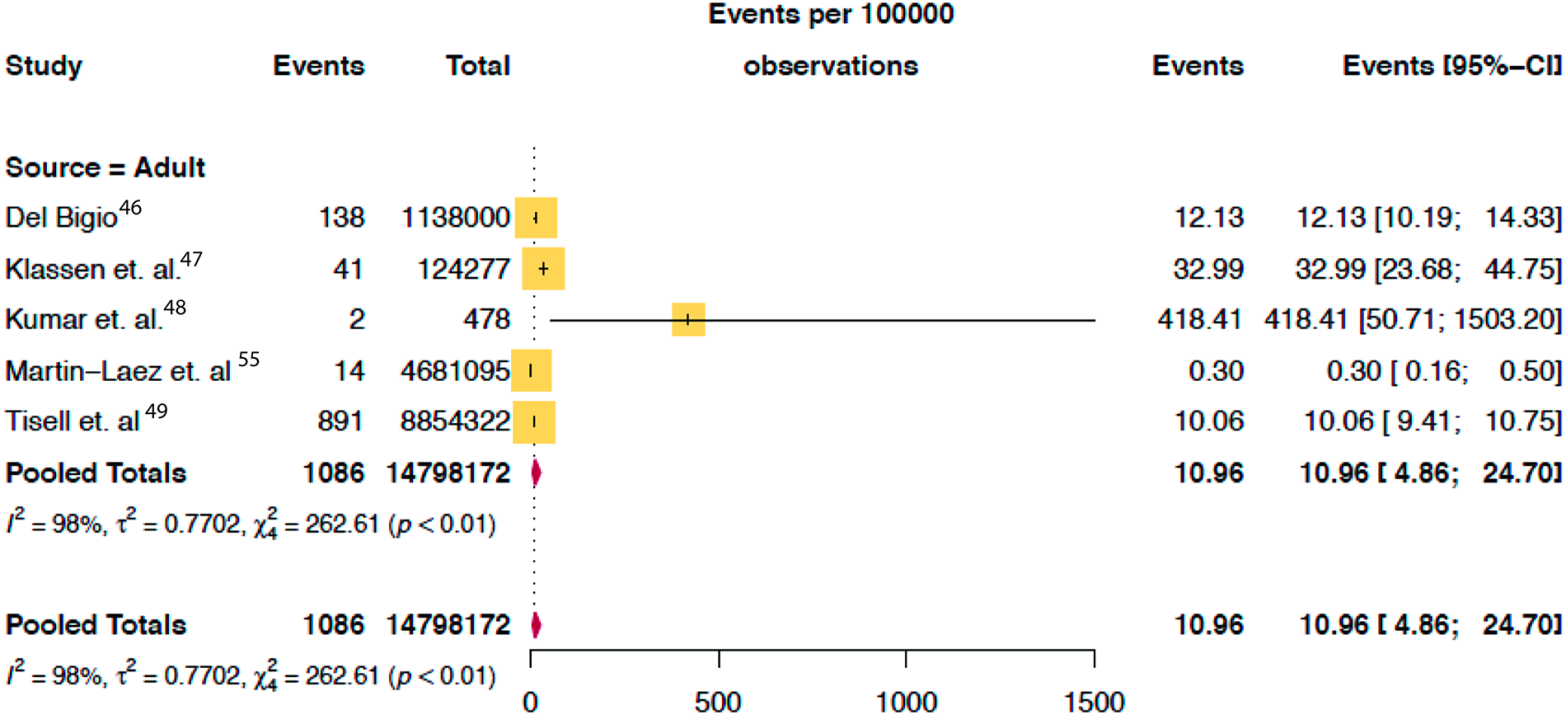
Pooled mean prevalence/100,000 of hydrocephalus in adult population.

**Fig 4:**
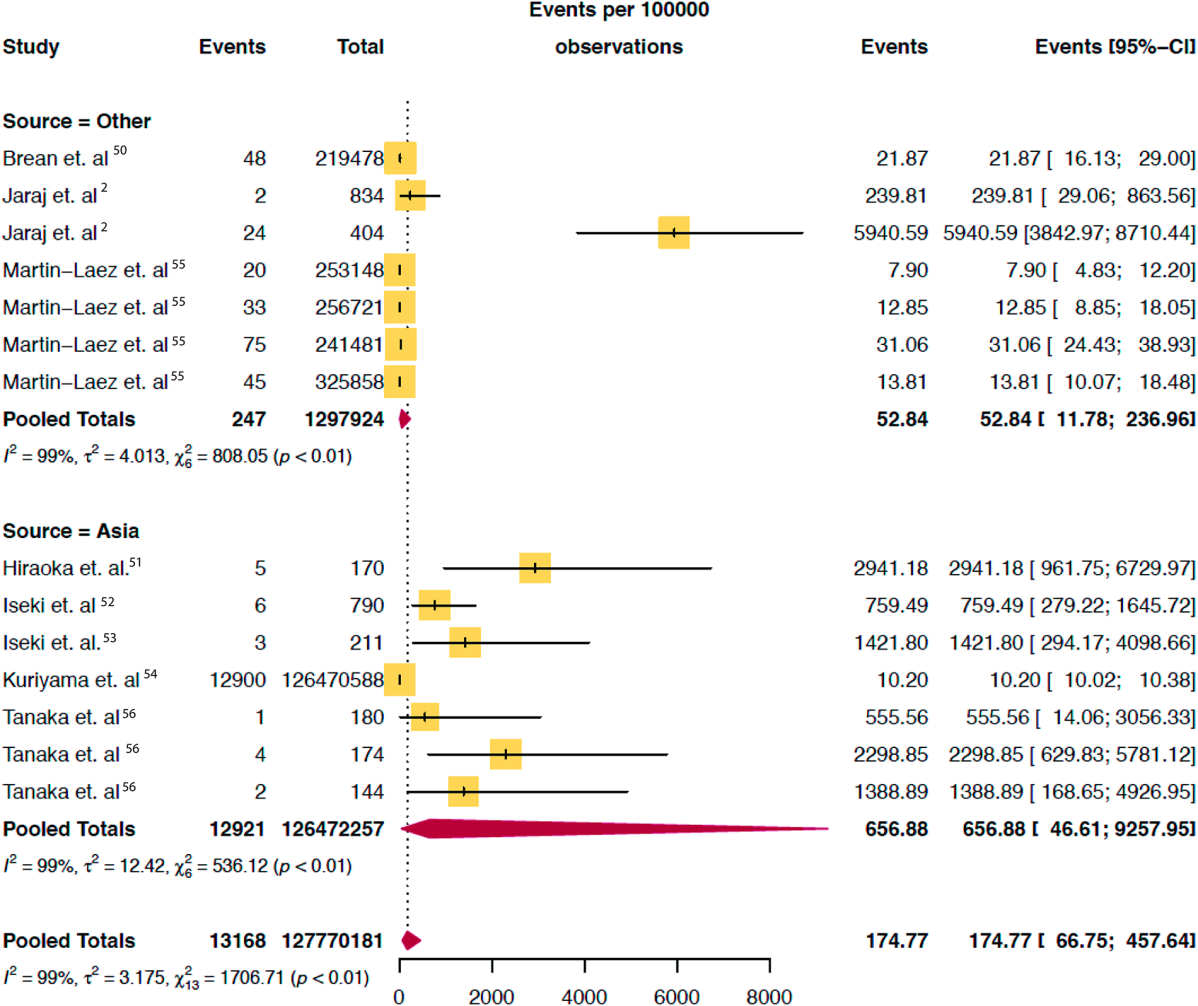
Pooled mean prevalence/100,000 of hydrocephalus in elderly population stratified by continent.

The combined search yielded 2,460 papers, of which 146 were selected for full text review. As shown in Fig 1, 52 studies met all eligibility criteria, two of which were identified via expert consultation. The total population assessed was 171,558,651 (28,990,298 pediatric, 14,798,172 adults and 127,770,181 elderly) as shown on Figs 2, 3 and 4.

### Prevalence of hydrocephalus (systematic review)

**Fig 5:**
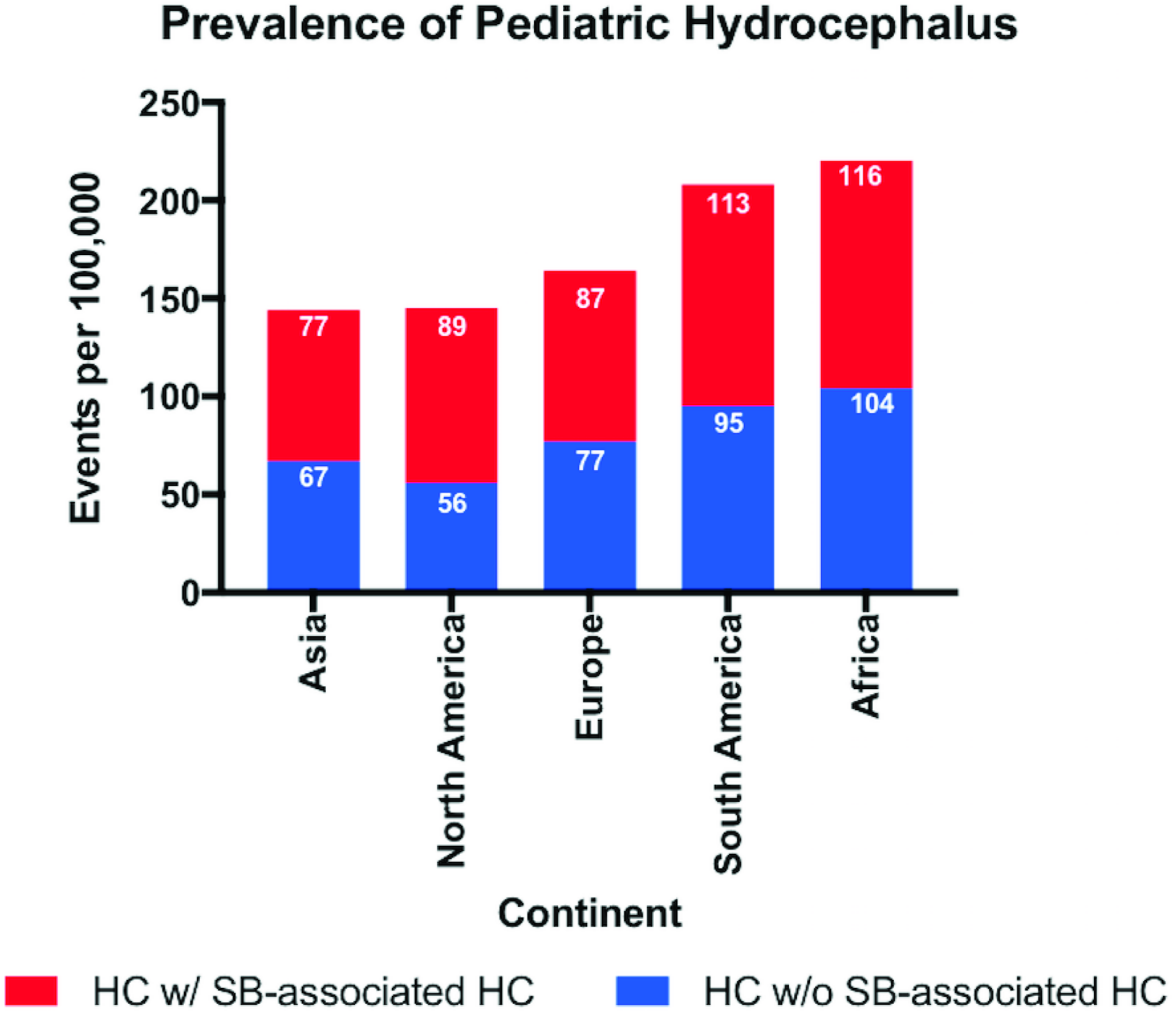
Prevalence (per 100,000) of pediatric hydrocephalus with (HC w/SB-associated HC) and without (HC w/o SB-associated HC) spina-bifida-associated hydrocephalus, stratified by continent.

**Fig 6:**
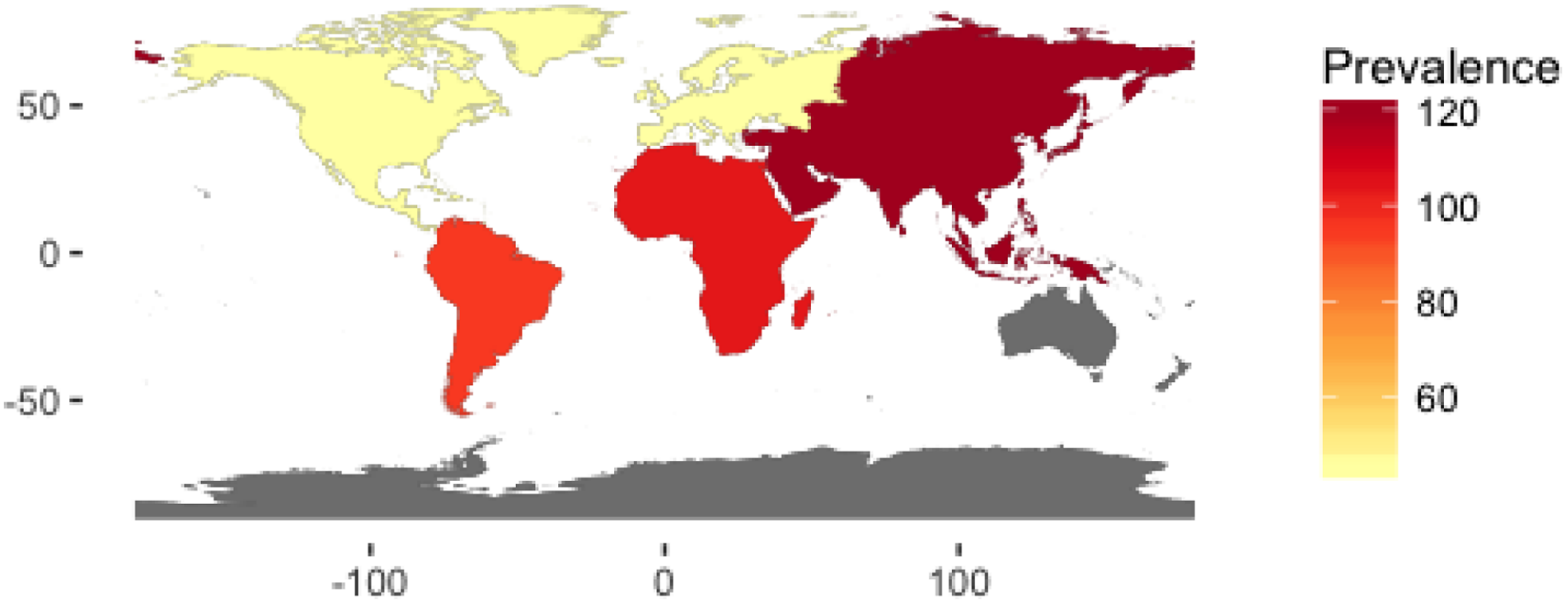
Prevalence (per 100,000) of hydrocephalus in the pediatric and elderly populations combined and shaded by continent from which the paper used in the meta-analysis was published.

Of the 52 studies reporting on the prevalence of hydrocephalus, 40 (77%) were in the pediatric population, 7 (13%) in the elderly population and 5 (10%) in adult population. The median study quality score was 5/8 (range 3–8). The overall global prevalence of hydrocephalus was 84.7/100,000 [95% CI 61.9 to 115.9]. The pooled prevalence of isolated hydrocephalus in the pediatric population was 71.9/100,000 [95% CI, 58.3 to 88·6]. When spina bifida-associated hydrocephalus was included, the prevalence increased to 87.8/100,000 [95% CI 71.9 to 107.3] (Fig 2). The prevalence of pediatric hydrocephalus between continents was almost two-fold higher in Africa (104.0/100,000 [95% CI 33.3 to 324.77]) compared with North America (55.6/100,000 [95% CI 41.4 to 74.7]) (Fig 5). Adults had the lowest reported prevalence of 10.9/100,000 [95% CI 4.9 to 24.7 (Fig 3). The highest prevalence was reported in the elderly at 174.8/100,000 [95% CI 66.8 to 457.6] (Fig 4). Heterogeneity existed between all estimates: pediatrics (*I*^*2*^ = 99.0% Q *p value* < 0.01), adults (*I*^*2*^ = 98.0% Q *p value* < 0.01) and elderly (*I*^*2*^ = 99.0% Q *p value* < 0.01). As demonstrated on Fig 4, the reported prevalence among the elderly population in Asia, 656.9/100,000 [95% CI 46.6 to 9257.9] was ten-fold that of Europe and North America combined, 52.8/100,000 [95% CI 11.8 to 237·0]. The prevalence has been represented on a world map shaded by continent where the population studied was based (Fig 6). The R-script used to generate the map had been provided as supplementary material (S3 Fig). Studies examining the elderly population reported prevalence stratified for age reported an increase in prevalence greater than 400/100,000 in the >80-year old group.[3] There was no significant difference in prevalence by the source of data collection among studies in all cohorts. Across the age continuum, the pooled prevalence was bimodal, with a nadir of the adult group. On visual inspection of the funnel plots or statistically with the Begg and Egger tests, there was no evidence of publication bias (all *p* > 0.05).

### Incidence of hydrocephalus (registry)

**Fig 7:**
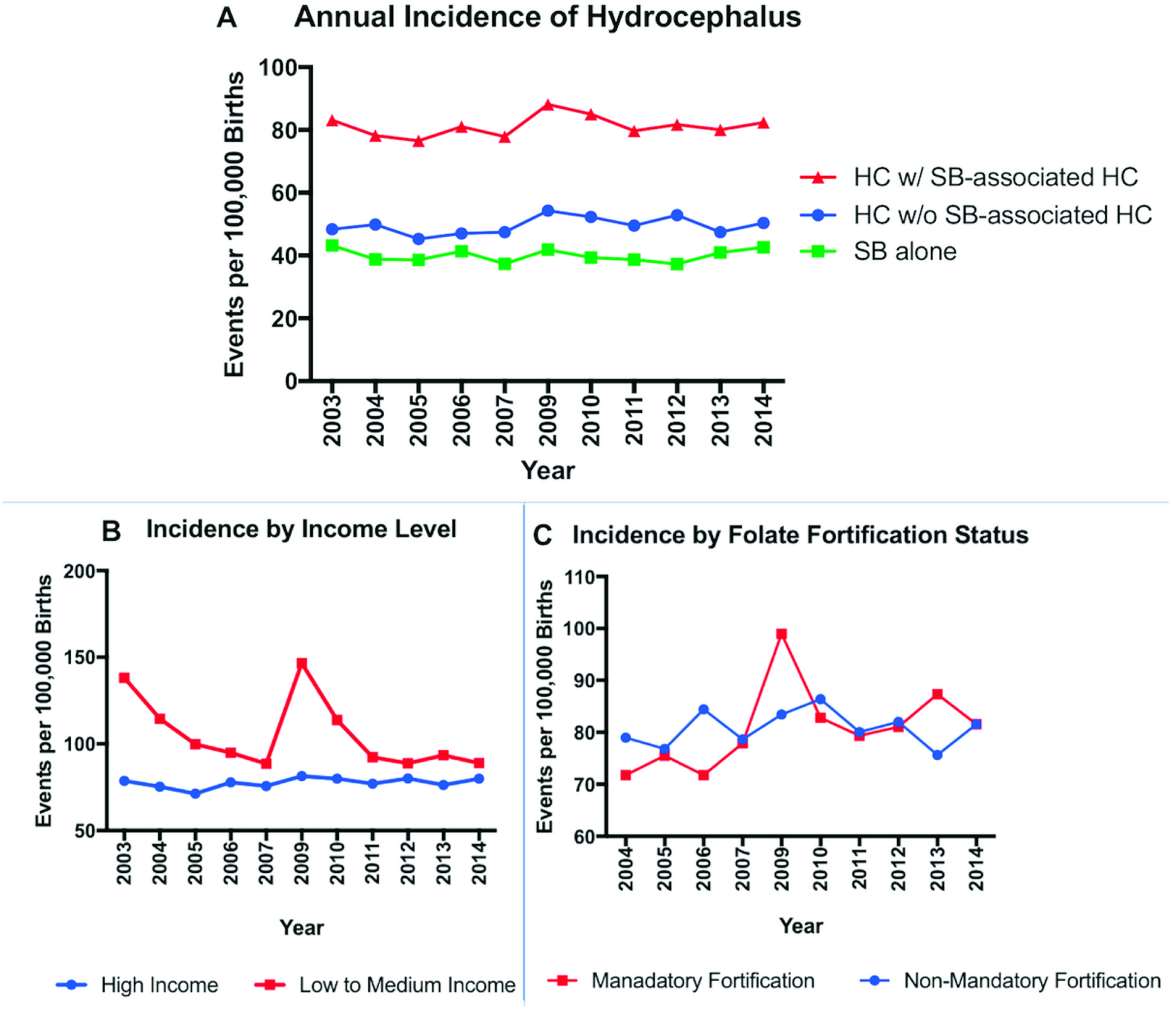
Annual incidence of perinatal hydrocephalus from 2003–2014. Image A demonstrates mean annual incidence in hydrocephalus with (HC w/SB-associated HC) and without (HC w/o SB-associated HC) Spina-bifida-associated hydrocephalus. The difference in mean annual incidence between high vs low/medium income (B) and between countries with and without mandatory folate fortification (C) are depicted.

The mean annual incidence of congenital hydrocephalus over the 11-year period (Fig 7A), was 49.5/100,000 [95% CI 41.1 to 59.8] for isolated hydrocephalus and 81.2/100,000 [95% CI 69.1 to 95.5] when spina-bifida associated hydrocephalus is factored in. As shown in Fig 7B, high income country level was associated with a significantly lower mean incidence of congenital hydrocephalus, 77.6/100,000 [95% CI 65.4 to 92.1], when compared to low- and middle-income countries combined, 105.5/100,000 [95% CI 76.1 to 147.6], *p* < 001. However, over the 11 years, there was no significant difference in mean incidence of congenital hydrocephalus between countries with and without mandatory folate fortification; 80.6/100,000 [95% CI 65.2 to 99.8] vs 80.9/100,000 [95% CI 65.5 to 99.2] respectively, *p* = 0·99 (Fig 7C). The mean incidence of spina bifida over the 11-year period was 40.0/100,000 [95% CI 33.5 to 47.9].

## Discussion

Hydrocephalus is a heterogeneous disease marked by abnormal dilatation of the cerebral ventricles secondary to varying etiologies[1]. This disease affects all age groups, from in-utero to old age,[2] and its prevalence and incidence are expected to rise with ageing demography. The mortality associated with untreated hydrocephalus is alarmingly high, ranging from to 20–87%.[71, 72] The morbidity associated with hydrocephalus is significant and includes seizures, developmental delay, psychomotor retardation, dementia and gait difficulties. At a health systems level, the diagnostic process and in-hospital costs associated with hydrocephalus management results in a high financial burden. Inpatient care of pediatric hydrocephalus patients alone a decade ago was reported to cost approximately $2 billion per year in the United States alone.[73] While already substantial, this does not account for the costs associated with out of hospital pediatric hydrocephalus care, or the costs for caring for the other age groups with hydrocephalus. Further, there is no effective medical therapy available to treat hydrocephalus. The only current treatment for hydrocephalus is surgical intervention typically with an implanted shunt system or in a limited patient subpopulation, an endoscopic third ventriculostomy (ETV).[74] With only 50% efficacy for shunts in the first two years after surgical placement, hydrocephalus continues to be a major global health problem, especially in countries with limited resources.[75] However, the lack of clarity regarding hydrocephalus epidemiology has negatively affected awareness and the proportionate allocation of resources to investigate and treat the disease.[76]

In this systematic review and meta-analysis of population-based epidemiological studies, we found an overall hydrocephalus global prevalence of 85/100,000. When stratified by age groups, the global prevalence of hydrocephalus is 88/100,000 in the pediatric population, 11/100,000 in adults and 175/100,000 in the elderly and potentially >400/100,000 in those >80 years of age. The prevalence of hydrocephalus is significantly higher in Africa and South America when compared to other continents.

From congenital birth defect registries, the incidence of hydrocephalus was 81/100,000 births. This would not identify postnatal causes of hydrocephalus which would be expected to result in an incidence of hydrocephalus that is higher by one year of age. Countries with lower income level had significantly higher incidence of congenital hydrocephalus. Similar trends have recently been reported in a review by Dewan et. al. (2018), which found an incidence of congenital hydrocephalus of 79 vs 123 per 100,000 births among low-and middle-income vs high income countries, respectively[6].

Although folate fortification is mandatory in many countries and numerous reviews have supported the use of folate as a prenatal or continuing supplement[77–83], the effect of folate supplementation on hydrocephalus (in humans) has not been well characterized. In fact, neither the original Medical Research Council (MRC) Vitamin Study Research Group trial[84] nor subsequent reports have adequately addressed the issue of hydrocephalus and folate supplementation[84–87]. However, given that approximately 80% of infants with spinal tube defects develop hydrocephalus[64, 65], one would expect a decrease in hydrocephalus, along with the reported decrease in spina bifida incidence with supplementation. While we did not find any difference in hydrocephalus incidence with or without mandatory folate fortification, we would caution against making any major inferences from these findings. It is important to recognize that the issue of folate fortification (even for spina bifida) is highly complex and controversial. In order to accurately inform patients, families and policy makers worldwide on the effect folate fortification on hydrocephalus, further studies are required. Nevertheless, this study may be leveraged to stimulate interest in future studies designed with a focused objective on the effect of mandatory folate fortification on the epidemiology of hydrocephalus.

The reported prevalence of hydrocephalus in adults in this study demonstrates a U-shaped pattern across the age continuum, with an 8-fold decline from pediatrics to adults and a subsequent 17-fold rise to the elderly. It is important to note that hydrocephalus is a chronic disease and the survival of pediatric hydrocephalus patients with surgical treatment is high.[88] As such, it is possible that a large proportion of adults with hydrocephalus might have stable disease from childhood and either tend not to seek medical attention or are under-reported by care providers. Therefore, prevalence by definition should include all patients with the diagnosis in the adult population, which also include patients who received treatment during childhood. This underscores the need for more research regarding health-related outcomes for children with hydrocephalus who transition into adulthood so that this prevalence information is captured. The bimodal pattern in estimates may also be partly attributed to “compensated/arrested hydrocephalus”, that has been hypothesized as a quiescence of congenital hydrocephalus during the pediatric-adult age transition, which later decompensates to resurface in the elderly age.[89] Interestingly, some forms of compensated hydrocephalus has been implicated in a subset of patients developing idiopathic normal pressure hydrocephalus (iNPH), a form of hydrocephalus which predominates in the elderly population.[90, 91] A few reports have attributed the reported high prevalence of elderly hydrocephalus (iNPH) to a trend of over-diagnosis or misdiagnosis of other forms of neurodegenerative diseases.[92, 93] While it is beyond the scope and deviates from the objectives of this study, there is no reliable evidence to support these claims.

This manuscript presents information regarding the global population-based epidemiology of hydrocephalus to better inform the healthcare community, policy makers and the public. There are however, specific nuances of hydrocephalus epidemiology outside of this structured analysis that also require attention. As previously mentioned, hydrocephalus is a heterogeneous disease that emanates from, as well as complicates a broad range of intracranial conditions such as trauma, infection, hemorrhage, tumors and genetic syndromes. Within these distinct subgroups of hydrocephalus etiologies, there is significant variation in the incidence and prevalence of hydrocephalus that is not easily captured by the methodology used for our prevalence evaluation. However, the diagnosis of hydrocephalus in these diagnoses significantly impact patient care and is also of critical importance to the healthcare provider.

Aneurysmal subarachnoid hemorrhage is a risk factor for developing both acute obstructive hydrocephalus and chronic communicating hydrocephalus. Our search strategy identified 9 papers reporting on the incidence of treated hydrocephalus in this population, which ranged from 10%[94] to 65%.[95] There is considerable inconsistency in reported shunt treatment rates which suggests a marked variability with respect to threshold for surgical treatment. Bekelis et al document the expected difference in shunting rates after endovascular coiling of 10,607 aneurysms, 6,056 of which were unruptured. Overall, 16.20% required shunting post-coiling: 36.67% in the ruptured aneurysm (subarachnoid hemorrhage (SAH)) group, and 0.83% in the unruptured group.[96] In a report by Hoh et al examining a nationwide inpatient database (2002–2007) of 6593 SAH patients who underwent aneurysm clipping and 4306 patients with SAH who underwent aneurysm coiling, only 9.25% and 10.54% (respectively) of the patients underwent shunt insertion.[94] More recently attempts have been made using multivariate analysis to identify risk factors for hydrocephalus requiring treatment after aneurysmal subarachnoid hemorrhage. These included volumes of CSF drainage in the convalescent period, higher SAH clinical grade, presence of acute hydrocephalus, intraventricular hemorrhage, re-hemorrhage, posterior circulation aneurysm, and age greater than 60 years.[97, 98] However, the dramatic variability in surgical treatment frequency for SAH-associated hydrocephalus implies that while SAH is a risk factor for development of hydrocephalus, there exists a need to better understand the best appropriate criteria for treatment.

Post-infectious hydrocephalus is a major global health problem, with high prevalence in Africa and Asia.[99–103] Systematic reviews of the prevalence of post-bacterial meningitis hydrocephalus have reported a prevalence of 6.80%.[104] While the pathogen may vary, the devastating effects of bacterial-associated hydrocephalus is uniform. Patients with community-acquired *Escherichia coli* and *Streptococcus pneumoniae* meningitis who develop post infectious hydrocephalus have a mortality risk of almost 60.00% compared to 17.00% for patients who have meningitis without hydrocephalus.[105]

Patients with brain tumors with or without surgical treatment also experience an increased risk of hydrocephalus. Pediatric posterior fossa tumors represent a subgroup at distinct risk for hydrocephalus at presentation as well as post-operative hydrocephalus that may require CSF diversion surgery. Prevalence of hydrocephalus at presentation can be as high as 87.18%.[106] Prevalence of post-operative hydrocephalus requiring permanent CSF diversion ranges from 21.53% in 130 consecutive patients with medulloblastoma[107] and 10.00–38.71% at long-term follow up in overall posterior fossa brain tumors in two pediatric patient populations.[108, 109] Less obviously, other tumors were also variably associated with hydrocephalus: supratentorial malignant glioma in adults, 10.00%,[110] giant pituitary adenoma, 8.33%,[111] and vestibular schwannoma, 15.16%.[112] Again, the variability in surgical treatment frequency for brain tumor-associated hydrocephalus implies that while brain tumor is a risk factor for development of hydrocephalus, the appropriate criteria for treatment are not clearly defined.

## Limitations and future directions

One of the drawbacks to combining these studies stems from the lack of consensus on a unifying definition or classification of hydrocephalus. While a working description of hydrocephalus has been proposed[1], the existing differing classification and definition of the disease, and the lack of standardization in epidemiological reporting practices precludes a robust analysis. The varying definition of hydrocephalus and methods of screening and diagnosis contributed to between study heterogeneity. However, despite this heterogeneity, these are the best possible estimates regarding the global epidemiology of hydrocephalus, which now sets the stage for future studies to unravel the vital questions surrounding the various subtypes of secondary hydrocephalus.

Of the 2,460 papers that we initially identified, 23 (0.9%) were excluded as they were published in languages other than English and French. A list of those papers has been provided on S2 Table. The possibility that our calculated prevalence of hydrocephalus may change if those excluded papers were added to the data analysis is small given that 21/23 of these papers dealt with congenital or infantile hydrocephalus and our analysis was based upon reports of almost 29 million pediatric patients. In addition, although this study utilized the ICBDSR to identify the global incidence of hydrocephalus, we recognize that there are other congenital birth defects surveillance programs such as the United States Center for Disease Control and the World Health Organization that may be utilized by future studies. A majority of the papers included in the prevalence and incidence analyses, respectively emanate from medium to high-income countries. Therefore, there is a possibility that the epidemiological data presented in this manuscript may be an underestimation due to under-notification in low income countries. However, these are not felt to be significant issues and we are confident in the precision of the prevalence and incidence of hydrocephalus in the pediatric population presented in this paper. The precision of the prevalence estimates for the elderly and more so the adult data were limited by the small number of high-quality population-based epidemiology papers that were available.

While the specific nuances of the epidemiology of secondary hydrocephalus such as those due to trauma, infection, hemorrhage, tumors and genetic syndromes have been previously reported[94–112], the majority of the existing literature is not population-based and therefore was not included in our structured analysis. However, hydrocephalus in these groups is also of critical importance and future studies may focus on addressing those.

## Conclusions

Hydrocephalus is a common neurologic condition that has significant implications for the patient and society. Previously, a lack of consistent epidemiological data has negatively affected the awareness of the disease and promoted incommensurate allocation of resources for the care of patients and research. We were able to estimate the global prevalence of hydrocephalus in pediatric, adult, and elderly populations and determine the global incidence of hydrocephalus. While folate fortification was not associated with the incidence of hydrocephalus, the incidence of hydrocephalus was higher in low-medium income compared to high-income countries. The expected increase in the elderly with aging demography, underscores the importance of healthcare resource allocation and further study of the burden of hydrocephalus.

## Supporting information

S1 Table Checklist items with corresponding page numbers for the PRISMA (2009) guidelines. (DOC)

S2 Table Citations in non-English or French language that were excluded during abstract reviews. (DOCX)

S1 Fig Search criteria for MEDLINE, EMBASE, Cochrane and Google Scholar databases. (PDF)

S2 Fig Data extraction form for systematic review. (PDF)

S3 Fig R script for generating world map shaded by continent with the prevalence of hydrocephalus in the pediatric and elderly populations[66]. (PDF)
