# Correction: Age-specific global epidemiology of hydrocephalus: Systematic review, metanalysis and global birth surveillance

**DOI:** 10.1371/journal.pone.0210851

**Published:** 2019-01-10

**Authors:** Albert M. Isaacs, Jay Riva-Cambrin, Daniel Yavin, Aaron Hockley, Tamara M. Pringsheim, Nathalie Jette, Brendan Cord Lethebe, Mark Lowerison, Jarred Dronyk, Mark G. Hamilton

The reference numbers within Figs 2, 3 and 4 are incorrect. Please see the corrected figures here.

**Fig 2:**
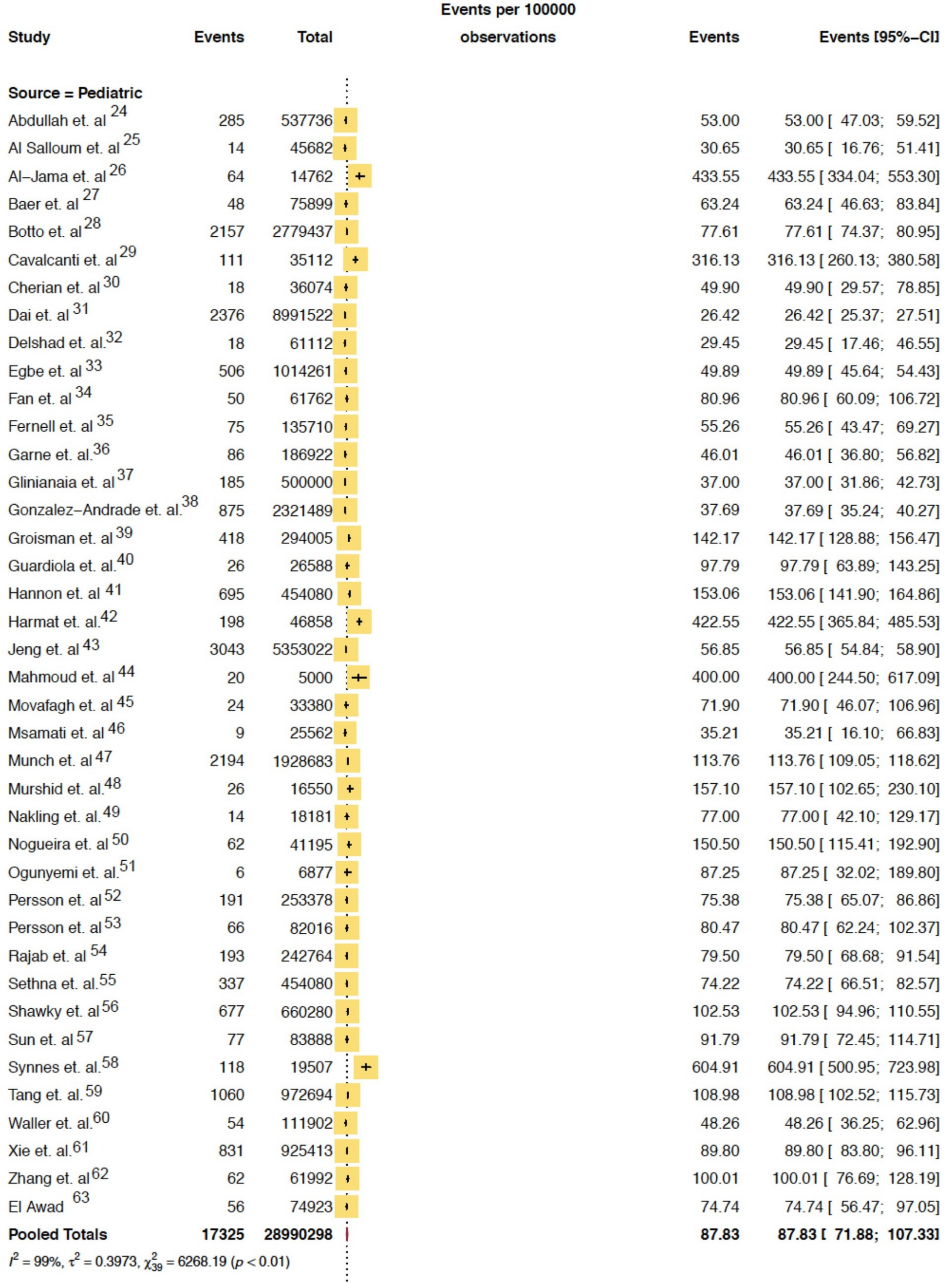
Pooled mean prevalence/100,000 of hydrocephalus in pediatric population.

**Fig 3:**
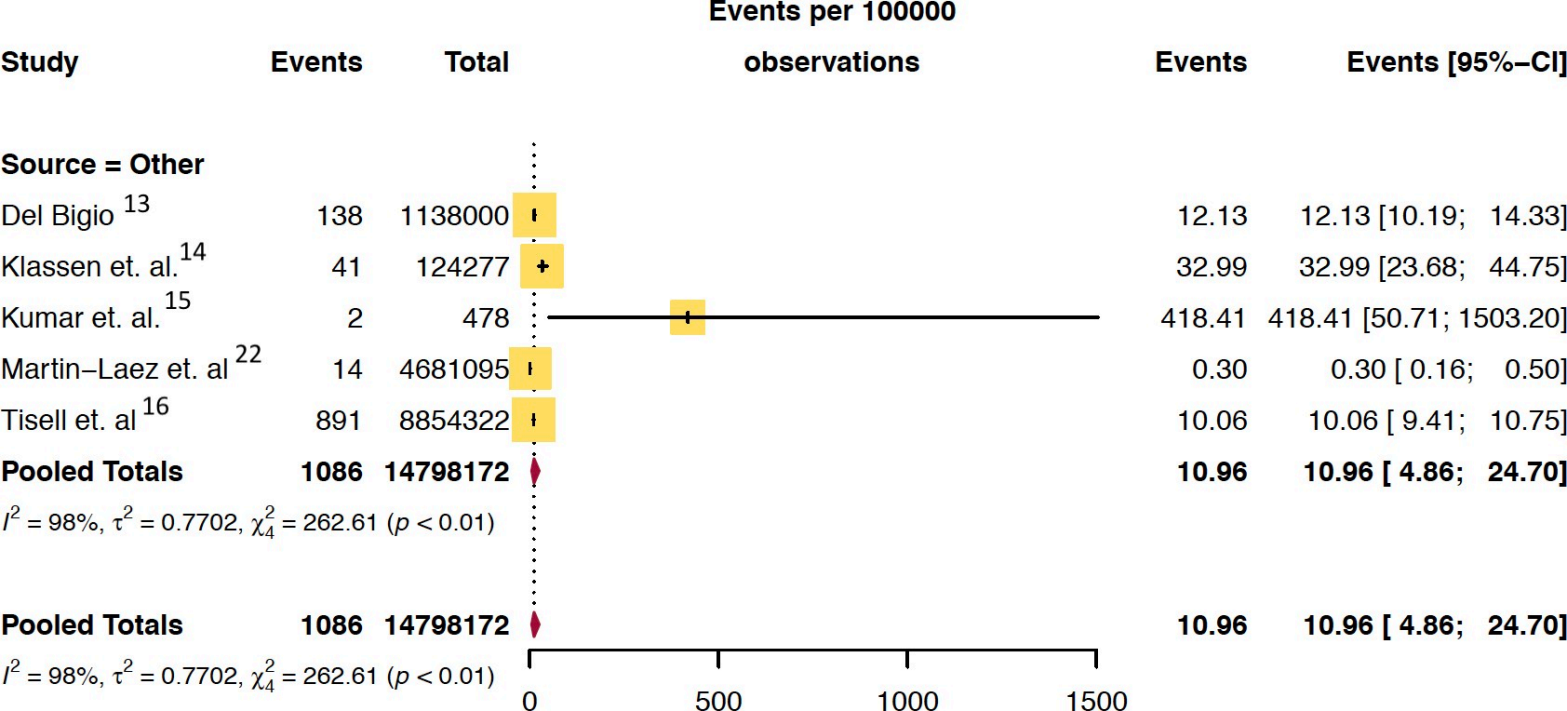
Pooled mean prevalence/100,000 of hydrocephalus in adult population.

**Fig 4:**
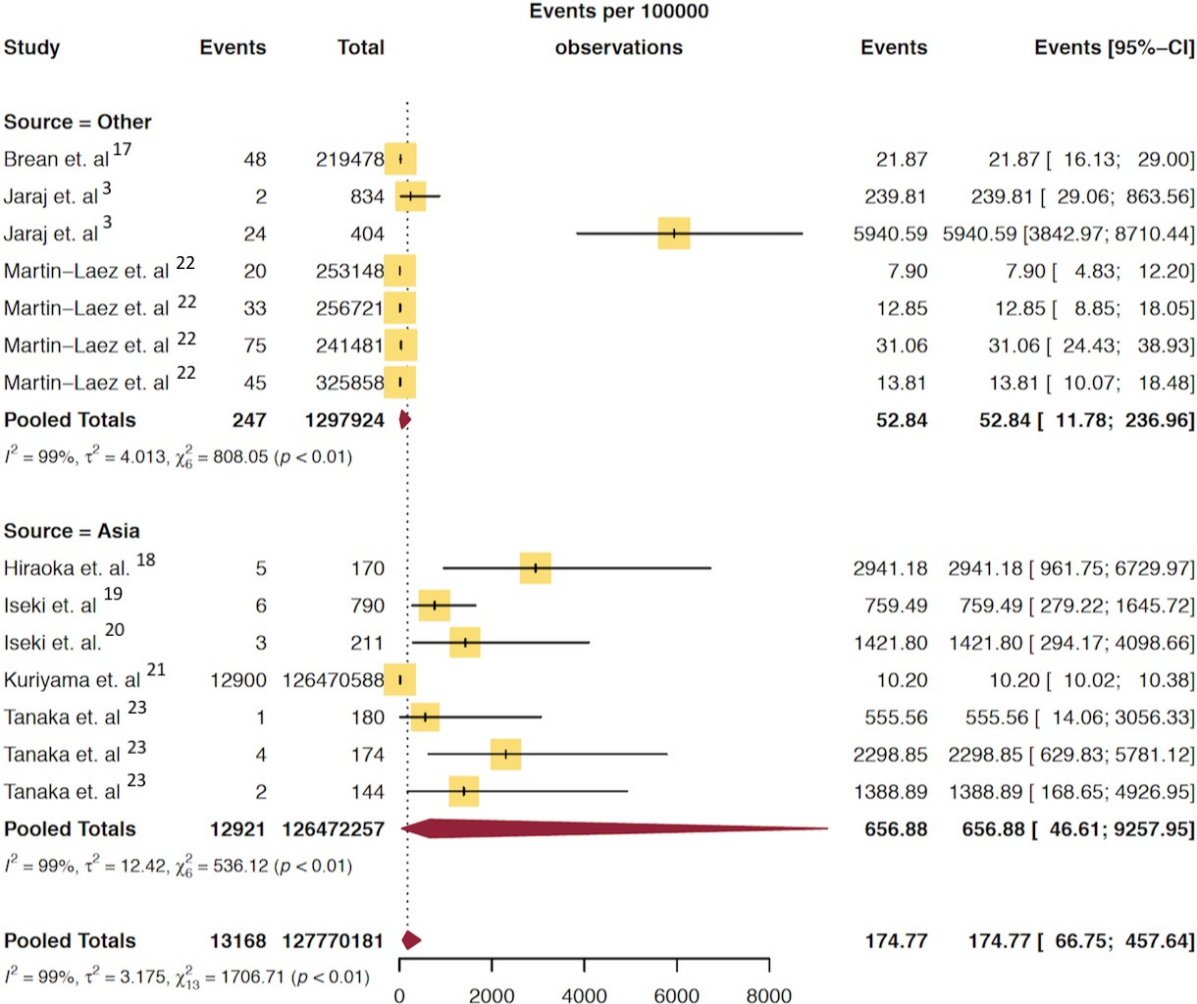
Pooled mean prevalence/100,000 of hydrocephalus in elderly population stratified by continent.
