# Supplementary material for: Age-specific global epidemiology of hydrocephalus: Systematic review, metanalysis and global birth surveillance
Source: PLoS One. 2018 Oct 1;13(10):e0204926. doi: 10.1371/journal.pone.0204926 (PMC6166961; doi:10.1371/journal.pone.0204926)
Supplement: S2 Table — (DOCX) [file pone.0204926.s002.docx]

**S2 Table:** Citations in non-English or French language that were excluded during abstract reviews.

|  | **First Author** | **Title** | **Citation** | **Language** |
| --- | --- | --- | --- | --- |
| 1 | Dai et. al.[1] | [Prevalence analysis on congenital hydrocephalus in Chinese perinatal from 1996 to 2004]. | Chung-Hua Yu Fang i Hsueh Tsa Chih [Chinese Journal of Preventive Medicine]. 40(3):180-3, 2006 May. | Chinese |
| 2 | Hu et. al.[2] | [A five years surveillance on neural system birth defects in rural areas of China]. | Chung-Hua Liu Hsing Ping Hsueh Tsa Chih Chinese Journal of Epidemiology. 17(1):20-4, 1996 Feb. | Chinese |
| 3 | Shi et. al.[3] | [Genetic epidemiologic study of hydrocephalus.] | Zhonghua fu chan ke za zhi. 25 (3) (pp 143-145, 187-188), 1990. Date of Publication: May 1990. | Chinese |
| 4 | Sipek et. al.[4] | [Congenital hydrocephalus 1961-2000--incidence, prenatal diagnosis and prevalence based on maternal age]. [Czech] | Ceska Gynekologie. 67(6):360-4, 2002 Nov. | Czech |
| 5 | Sipek et. al.[5] | [Incidence and survival in children with selected types of congenital defects in the Czech Republic from 1994 to 2001. (Part 1)]. | Ceska Gynekologie. 69(1):59-65, 2004 Jan. | Czech |
| 6 | Sipek et. al.[6] | [Occurrence of congenital hydrocephalus in the Czech Republic 1961-1995.] | Ceska gynekologie / Ceska lekarska spolecnost J. Ev. Purkyne. 63 (3) (pp 207-210), 1998. Date of Publication: Jun 1998. | Czech |
| 7 | Christensen et. al.[7] | [Congenital hydrocephalus-prevalence and prognosis. Mortality and morbidity in a population-based study]. | Ugeskrift for Laeger. 165(5):466-9, 2003 Jan 27. | Danish |
| 8 | den Quden et. al.[8] | [Prevalence, clinical aspects and prognosis of neural tube defects in The Netherlands]. | Nederlands Tijdschrift voor Geneeskunde. 140(42):2092-5, 1996 Oct 19. | Dutch |
| 9 | Robroch et. al.[9] | [Ventriculomegaly at the gestational age of 20 weeks; research into its incidence and related abnormalities]. | Nederlands Tijdschrift voor Geneeskunde. 157(3):A5148, 2013. | Dutch |
| 10 | Langmar et. al.[10] | [Congenital disorders. Hydrocephalus]. | Orvosi Hetilap. 152(52):2098-102, 2011 Dec 25. | Hungarian |
| 11 | Kato et. al.[11] | [iNPH (Idiopathic normal pressure hydrocephalus) and AVIM (asymptomatic ventriculomegaly with features of iNPH on MRI)]. | Rinsho Shinkeigaku - Clinical Neurology. 50(11):963-5, 2010 Nov. | Japanese |
| 12 | Negoro et. al.[12] | [Clinico-epidemiologic study of infantile hydrocephalus in Japan]. | No to Hattatsu [Brain & Development]. 26(3):211-5, 1994 May. | Japanese |
| 13 | Suzuki et. al.[13] | [Occurrence of anomalies of the central nervous system in handicapped children]. | No to Hattatsu [Brain & Development]. 24(1):9-13, 1992 Jan. | Japanese |
| 14 | Sobaniec-Lotowska et. al. [14] | [Congenital anomalies of the central nervous system in autopsy specimens]. | Wiadomosci Lekarskie. 42(3):159-63, 1989 Feb 01. | Polish |
| 15 | Cavalcanti et. al.[15] | [Incidence of congenital hydrocephalus and the role of the prenatal diagnosis]. | Jornal de Pediatria. 79(2):135-40, 2003 Mar-Apr. | Portuguese |
| 16 | Kliemann et. al.[16] | [Shunted hydrocephalus in childhood: an epidemiological study of 243 consecutive observations]. | Arquivos de Neuro-Psiquiatria. 63(2B):494-501, 2005 Jun. | Portuguese |
| 17 | Chiosac et. al.[17] | [Research on the incidence and prevalence of congenital abnormalities in Iasi district and Iasi city, from 2001 to 2008]. | Revista medico-chirurgicala a Societatii de Medici si Naturalisti din Iasi. 114 (3) (pp 836-840), 2010. Date of Publication: 2010 Jul-Sep. | Romanian |
| 18 | Kayuka et. al.[18] | [The dynamics of the incidence of intraventricular hemorrhages and their outcomes among prematurely born children of the poltava region in 2007-2016]. | Wiadomosci Lekarskie. 70(3 pt 1):493-498, 2017. | Russian |
| 19 | Grillo et. al.[19] | [Neural tube defects and congenital hydrocephalus. Why is prevalence important?]. | Jornal de Pediatria. 79(2):105-6, 2003 Mar-Apr. | Spanish |
| 20 | Perez et. al.[20] | [Ultrasound diagnosis of fetal ventriculomegaly. Postnatal follow-up.] | Progresos de Obstetricia y Ginecologia. 57 (5) (pp 202-207), 2014. Date of Publication: May 2014. | Spanish |
| 21 | Ruggieri et. al.[21] | [Genetic syndromes recognizable in the neonatal period.] | Medicina. 69 (1 PART 1) (pp 15-35), 2009. Date of Publication: 2009. | Spanish |
| 22 | Fernell et. al.[22] | [Infantile hydrocephalus. Complication of prematurity]. | Lakartidningen. 90(9):791-3, 1993 Mar 03. | Swedish |
| 23 | Hoglund et. al.[23] | [Incidence of surgery for hydrocephalus in adults surveyed: same number afflicted by hydrocephalus as by multiple sclerosis]. | Lakartidningen. 98(14):1681-5, 2001 Apr 04. | Swedish |

# **References**

1. Dai L, Zhou GX, Miao L, Zhu J, Wang YP, Liang J. [Prevalence analysis on congenital hydrocephalus in Chinese perinatal from 1996 to 2004]. Zhonghua Yu Fang Yi Xue Za Zhi. 2006;40(3):180-3. Epub 2006/07/14. PubMed PMID: 16836884.

2. Hu YH, Li LM, Li P. [A five years surveillance on neural system birth defects in rural areas of China]. Zhonghua Liu Xing Bing Xue Za Zhi. 1996;17(1):20-4. Epub 1996/02/01. PubMed PMID: 8758414.

3. Shi MA, Chen YL. [Genetic epidemiologic study of hydrocephalus]. Zhonghua Fu Chan Ke Za Zhi. 1990;25(3):143-5, 87-8. Epub 1990/05/01. PubMed PMID: 2390871.

4. Sipek A, Gregor V, Horacek J, Masatova D. [Congenital hydrocephalus 1961-2000--incidence, prenatal diagnosis and prevalence based on maternal age]. Ceska Gynekol. 2002;67(6):360-4. Epub 2003/03/29. PubMed PMID: 12661376.

5. Sipek A, Gregor V, Horacek J, Masatova D, Svetnicova K. [Incidence and survival in children with selected types of congenital defects in the Czech Republic from 1994 to 2001. (Part 1)]. Ceska Gynekol. 2004;69(1):59-65. Epub 2004/04/29. PubMed PMID: 15112390.

6. Sipek A, Gregor V, Horacek J, Chudobova M, Korandova V, Skibova J. [Occurrence of congenital hydrocephalus in the Czech Republic 1961-1995]. Ceska Gynekol. 1998;63(3):207-10. Epub 1998/09/29. PubMed PMID: 9750382.

7. Christensen JH, Hansen LK, Garne E. [Congenital hydrocephalus--prevalence and prognosis. Mortality and morbidity in a population-based study]. Ugeskr Laeger. 2003;165(5):466-9. Epub 2003/02/26. PubMed PMID: 12599846.

8. den Quden AL, Hirasing RA, Buitendijk SE, de Jong-van den Berg LT, de Walle HE, Cornel MC. [Prevalence, clinical aspects and prognosis of neural tube defects in The Netherlands]. Ned Tijdschr Geneeskd. 1996;140(42):2092-5. Epub 1996/10/19. PubMed PMID: 8965952.

9. Robroch B, Holwerda J, Bos AF, Bilardo CM, van den Berg PP, Snijders RJ. [Ventriculomegaly at the gestational age of 20 weeks; research into its incidence and related abnormalities]. Ned Tijdschr Geneeskd. 2013;157(3):A5148. Epub 2013/01/19. PubMed PMID: 23328015.

10. Langmar Z, Nemeth M, Csaba A, Beke A, Joo JG. [Congenital disorders. Hydrocephalus]. Orv Hetil. 2011;152(52):2098-102. Epub 2011/12/14. doi: 10.1556/OH.2011.29261. PubMed PMID: 22155518.

11. Kato T, Iseki C, Takahashi Y, Wada M, Kawanami T, Sato H, et al. [iNPH (Idiopathic normal pressure hydrocephalus) and AVIM (asymptomatic ventriculomegaly with features of iNPH on MRI)]. Rinsho Shinkeigaku. 2010;50(11):963-5. Epub 2011/09/17. PubMed PMID: 21921528.

12. Negoro T, Watanabe K, Nakashima S, Kikuchi H, Tamakoshi A. [Clinico-epidemiologic study of infantile hydrocephalus in Japan]. No To Hattatsu. 1994;26(3):211-5. Epub 1994/05/01. PubMed PMID: 8185972.

13. Suzuki H, Hirano S, Kurokawa T. [Occurrence of anomalies of the central nervous system in handicapped children]. No To Hattatsu. 1992;24(1):9-13. Epub 1992/01/01. PubMed PMID: 1531019.

14. Sobaniec-Lotowska M, Ostapiuk H, Sulkowski S, Sobaniec W, Sulik M, Famulski W. [Congenital anomalies of the central nervous system in autopsy specimens]. Wiad Lek. 1989;42(3):159-63. Epub 1989/02/01. PubMed PMID: 2623859.

15. Cavalcanti DP, Salomao MA. [Incidence of congenital hydrocephalus and the role of the prenatal diagnosis]. J Pediatr (Rio J). 2003;79(2):135-40. Epub 2003/09/23. PubMed PMID: 14502334.

16. Kliemann SE, Rosemberg S. [Shunted hydrocephalus in childhood: an epidemiological study of 243 consecutive observations]. Arq Neuropsiquiatr. 2005;63(2B):494-501. Epub 2005/08/02. doi: /S0004-282X2005000300024. PubMed PMID: 16059605.

17. Chiosac AA, Manole A, Gorduza EV, Stamatin M, Titianus M, Ivan A. [Research on the incidence and prevalence of congenital abnormalities in Iasi district and Iasi city, from 2001 to 2008]. Rev Med Chir Soc Med Nat Iasi. 2010;114(3):836-40. Epub 2011/01/20. PubMed PMID: 21243811.

18. capital Ka CaCeCyCzCkCaCcIECAC, capital A CeCtCiCeCoCvCaCcENCESC, capital Ghe CaCeCyCkCcENCIC, capital Tse CvCiCeCiCeCkCoCcESCENC, capital Pe CoChCiCeCssCkCoCcVECIC. [The dynamics of the incidence of intraventricular hemorrhages and their outcomes among prematurely born children of the poltava region in 2007-2016]. Wiad Lek. 2017;70(3 pt 1):493-8. Epub 2017/07/18. PubMed PMID: 28711895.

19. Grillo E, da Silva RJ. [Neural tube defects and congenital hydrocephalus. Why is prevalence important?]. J Pediatr (Rio J). 2003;79(2):105-6. Epub 2003/09/23. PubMed PMID: 14502329.

20. Pina Pérez S, Costa Pueyo J, Serra Azuara L, Molina Marín C, Escofet Soteras C, Corona Martínez M. Diagnóstico ecográfico de la ventriculomegalia fetal. Seguimiento posnatal. Progresos de Obstetricia y Ginecología. 2014;57(5):202-7. doi: 10.1016/j.pog.2014.01.007.

21. Ruggieri VL, Arberas CL. [Genetic syndromes recognizable in the neonatal period]. Medicina (B Aires). 2009;69(1 Pt 1):15-35. Epub 2009/02/26. PubMed PMID: 19239999.

22. Fernell E, Hagberg G, Hagberg B. [Infantile hydrocephalus. Complication of prematurity]. Lakartidningen. 1993;90(9):791-3. Epub 1993/03/03. PubMed PMID: 8445965.

23. Hoglund M, Tisell M, Wikkelso C. [Incidence of surgery for hydrocephalus in adults surveyed: same number afflicted by hydrocephalus as by multiple sclerosis]. Lakartidningen. 2001;98(14):1681-5. Epub 2001/05/31. PubMed PMID: 11379170.
