# Supplementary material for: Age-specific global epidemiology of hydrocephalus: Systematic review, metanalysis and global birth surveillance
Source: PLoS One. 2018 Oct 1;13(10):e0204926. doi: 10.1371/journal.pone.0204926 (PMC6166961; doi:10.1371/journal.pone.0204926)
Supplement: S1 Fig — (PDF) [file pone.0204926.s003.pdf]

S1 Fig: Search criteria for MEDLINE, EMBASE, Cochrane and Google Scholar databases

A: MEDLINE Search

1. exp Incidence/
2. exp Prevalence/
3. exp Epidemiology/
4. 1 or 2 or 3
5. exp Hydrocephalus/
6. exp Hydrocephalus, Normal Pressure/
7. 4 and 5
8. 4 and 6
9. exp Hydrocephalus/ep [Epidemiology]
10. exp Hydrocephalus, Normal Pressure/ep [Epidemiology]
11. 7 or 8 or 9 or 10
12. limit 11 to yr="1985 -Current"
13. limit 12 to animals
14. 12 not 13

B: EMBASE search

1. exp incidence/
2. exp prevalence/
3. *epidemiology/
4. 1 or 2 or 3
5. exp congenital hydrocephalus/ or exp communicating hydrocephalus/ or exp normotensive hydrocephalus/ or exp hydrocephalus/
6. 4 and 5
7. exp congenital hydrocephalus/ep [epidemiology]
8. exp communicating hydrocephalus/ep [epidemiology]
9. exp normotensive hydrocephalus/ep [epidemiology]
10. exp hydrocephalus/ep [epidemiology]
11. 6 or 7 or 8 or 9 or 10
12. Limit 11 to yr="1985 -current"
13. Limit 12 to (animals and animal studies)
14. 12 not 13

C: Cochrane Database search

1. exp Incidence/
2. exp Prevalence/
3. exp Epidemiology/
4. 1 or 2 or 3
5. exp Hydrocephalus/
6. exp Hydrocephalus, Normal Pressure/
7. 4 and 5
8. 4 and 6
9. exp Hydrocephalus/ep [Epidemiology]
10. exp Hydrocephalus, Normal Pressure/ep [Epidemiology]
11. 7 or 8 or 9 or 10
12. limit 11 to yr="1985 -Current"

D: Google Scholar search

1. intitle:"hydrocephalus prevalence" OR ""hydrocephalus incidence" OR ""hydrocephalus epidemiology"
2. Custom range: 1985-2017
