# Supplementary material for: Age-specific global epidemiology of hydrocephalus: Systematic review, metanalysis and global birth surveillance
Source: PLoS One. 2018 Oct 1;13(10):e0204926. doi: 10.1371/journal.pone.0204926 (PMC6166961; doi:10.1371/journal.pone.0204926)
Supplement: S2 Fig — (PDF) [file pone.0204926.s004.pdf]

S2 Fig: Data extraction form for systematic review

NHCC-PHAC Systematic Review Data Collection Form

Reference Information: **REFERENCE ID #:**

Authors:

Title:

Journal:

Year: Volume: Issue: Page Start: Page End:

Study Population:

Population-Based Sample: ☐ Yes ☐ No (*exclude if answer is no)*

Original Data (i.e. not a review article) ☐ Yes ☐ No (*exclude if answer is no)*

Location of Population (e.g. city, province, state):

Continent Where Data Collection Occurred (check all that apply)

☐ Africa ☐ Asia
☐ Australia ☐ Europe
☐ North America ☐ South America

Time Period for Data Collection:
Exclude if data was collected prior to 1985

Sample Size:
Number surveyed:

Number of cases:

Response Rate: ☐ Not Applicable*
**Choose not applicable in studies that did not directly contact patients (i.e. chart reviews, administrative data studies, etc)*

Population Age
Range:

Mean:

Median:

Population Groups Studied (check all that apply)

☐ Fetuses ☐ Infants (<1 year)
☐ Children/Youth (1-18 years) ☐ Adults (19-64 years)
☐ Elderly (≥ 65 years)

DRAFT

Condition Information:

Definition of condition:

Data Sources (check all that apply):

- ☐ Door-to-door survey
- ☐ Telephone survey
- ☐ Mailed survey
- ☐ Administrative databases
- ☐ Hospital/clinic chart review
- ☐ Prescription drug database
- ☐ Registry
- ☐ Other (specify):

How was Diagnosis Established (check all that apply)?

- ☐ Clinical Assessment by a Health Professional
- ☐ Medical chart review
- ☐ Administrative data codes (specify codes:
- ☐ Self-report of a condition (diagnosed by a health professional)
- ☐ Self-report of a condition (undiagnosed by a health professional)
- ☐ Use of specific prescription drug
- ☐ Genetic test
- ☐ Imaging Test (ultrasound, CT, MRI, etc)
- ☐ Cannot determine from article
- ☐ Other (specify):

Were Diagnostic Criteria Used to Make the Diagnosis? ☐ Yes ☐ No ☐ Unclear

If yes, please list the criteria below (e.g. DSM-IV criteria for dementia, MacDonald Criteria for multiple sclerosis, etc):

Overall Incidence:

Overall Prevalence:

DRAFT

Breakdown of Incidence/Prevalence by Socio-Demographic Factors

		Incidence	Prevalence	Not Specified
Gender	Male			+
	Female			+
Area of Residence	Urban			+
	Rural			+
Age				+
Ethnicity				+

Quality Assessment [9, 10]

Articles are assigned 1 point for each "yes" received on the following scale for a total score out of 8
If a question is not applicable for the study under review, select "yes".

REPRESENTATIVENESS OF SAMPLE

1. Is the target population clearly defined? Select an option: **--------**

e.g. The target population must be defined by shared characteristics assessed and measure accurately. Some of these characteristics include age, sex, ethnicity, income, etc. Clear inclusion and exclusion criteria.

2. Was either of the following ascertainment method used (must be one or the other)

1) **probability sampling OR**
2) **entire population surveyed** Select an option: **--------**

e.g. Members of the target population were identified through a sampling frame or listing of potential respondents. This listing must provide access to all members or the defined target population except for exclusions acknowledged by the study authors.

3. Is the response rate ≥70% Select an option: **--------**

4. Are non-responders clearly described? Select an option: **--------**

DRAFT

5. Is the sample representative of the target population?

Select an option: **--------**

e.g. need to ensure that non responders have similar characteristics as responders (otherwise may have selection bias)

ASSESSMENT OF NEUROLOGICAL CONDITION

6. Were data collection methods standardized? Select an option: **--------**

e.g. Identical methods of assessment and data collection were used with all respondents so that the information for analysis is completely comparable. Standardization of methods not only refer to eliciting information from respondents but also to interviewing training, supervision, enlistment of respondents and processing of data.

7. Were validated criteria used to assess for the presence/absence of disease? Select an option: **--------**

e.g. or a validated scale, diagnostic tool, survey, etc.

STATISTICAL ANALYSIS

8. Are the estimates of prevalence and incidence given with confidence intervals and in detail by subgroup (if applicable)? Select an option: **--------**

TOTAL QUALITY SCORE: /8

DRAFT
