# Supplementary material for: Age-specific global epidemiology of hydrocephalus: Systematic review, metanalysis and global birth surveillance
Source: PLoS One. 2018 Oct 1;13(10):e0204926. doi: 10.1371/journal.pone.0204926 (PMC6166961; doi:10.1371/journal.pone.0204926)
Supplement: S3 Fig — (PDF) [file pone.0204926.s005.pdf]

```r
library(rgdal)
library(rgeos)
library(ggplot2)
library(maptools)
library(rworldmap)
library(httr)
library(dplyr)

url <- "https://gist.githubusercontent.com/hrbrmstr/91ea5cc9474286c72838/raw/f3fde312c9b816dff3994f39f2bcda03209eff8f/
    continents.json"
stop_for_status(GET(url, write_disk("continents.json",overwrite=TRUE)))
continents <- readOGR("continents.json", "OGRGeoJSON")
continents_map <- fortify(continents, region="CONTINENT")

#### For Specific Countries
w <- getMap()
## we need to id to join back
w$id_1 <- row.names(w)
world <- fortify(w)

continents_map <- world %>% inner_join(as.data.frame(w)[, c("ADMIN", "id_1")], c("id" = "id_1"))

#ggplot(world) + aes(x = long, y = lat, group = group, fill = ADMIN) + geom_polygon() + guides(fill = FALSE)
#####


data <- read.csv("~/Desktop/incidence.csv", header=TRUE, stringsAsFactors=FALSE)
colnames(data) = c("id","Prevalence")
data[,1] = trimws(data[,1])

data = left_join(continents_map,data)

gg <- ggplot()
gg <- gg + geom_map(data=data,
                    map=data,
                    aes(x=long, y=lat, map_id=id))

gg <- gg + geom_map(data=data,
                    map=continents_map,
                    aes(map_id=id, fill=Prevalence))

gg <- gg + scale_fill_gradientn(
  colours = ( brewer.pal( 7, "YlOrRd" ) )
)
gg <- gg + coord_equal()
gg <- gg + theme_bw()
gg <- gg + labs(x=NULL, y=NULL)
gg <- gg + theme(panel.border=element_blank())
gg <- gg + theme(panel.grid=element_blank())
gg
```
